# Peripheral Ameloblastoma: A Case Report and Review of Literature

**DOI:** 10.3390/jcm13226714

**Published:** 2024-11-08

**Authors:** Sem Decani, Martina Quatrale, Veronica Caria, Laura Moneghini, Elena Maria Varoni

**Affiliations:** 1 Department of Biomedical, Surgical and Dental Sciences, University of Milan, 20142 Milan, Italy; sem.decani@unimi.it (S.D.); martina.quatrale@studenti.unimi.it (M.Q.); 2 Odontostomatology II, San Paolo Hospital, ASST Santi Paolo e Carlo, 20142 Milan, Italy; veronica.caria@studenti.unimi.it; 3 Pathological Anatomy, San Paolo Hospital, ASST Santi Paolo e Carlo, 20143 Milan, Italy; laura.moneghini@asst-santipaolocarlo.it

**Keywords:** peripheral ameloblastoma (PA), extraosseous lesion, benign tumor

## Abstract

Peripheral ameloblastoma (PA) is a rare benign tumor that can occur mostly in the mandibular gingiva of the premolar area, originating from the residual odontogenic epithelium. The patient is usually asymptomatic and the lesion can be an occasional finding during routine intraoral examination. Due to the lack of clinical and radiographic pathognomonic features, the diagnosis is based on histopathological analysis, associated with 3D computed tomography (CT) imaging. Here, we report the case of a middle-aged man showing an asymptomatic, sessile, normochromic papillomatous mass of the lingual alveolar mucosa, in correspondence of tooth 4.4, which was histologically diagnosed as peripheral ameloblastoma. After the complete excision of the lesion, there were no recurrence and no symptoms during the 3-year follow-up. The treatment of choice for PA is a conservative surgical excision, which usually results in a good prognosis, together with a long-term follow-up, necessary to intercept possible recurrence or, more rarely, malignant transformation.

## 1. Introduction

In the oral cavity, the most common neoplasm that arises from the residual odontogenic epithelium is ameloblastoma. Although rare, accounting for about 1% of all jaw tumors [1,2], they represent the second-most common odontogenic tumor, with a pooled incidence rate of 0.92 per million person-years [3]. The term “ameloblastoma” originates from the early English “amel”, which means enamel, and the Greek word “blastos”, meaning germ. They are composed of the epithelium of ectodermal origin, deriving from the cells localized at the tooth root, or in close approximation, resulting from the ectoderm germ layer. Most ameloblastomas are benign and slow-growing, with locally aggressive behavior. The patients may experience no symptoms until the tumor is of a larger size. Signs most commonly reported are as follows: abnormal growth in the jaw or sinus area, painless swelling in the jaw, delayed tooth eruption. The abnormal cell growth easily infiltrates local tissue, typically bone. Surgical excision is usually needed to treat this disorder, which has a high propensity for local recurrence even with an appropriate surgical management and requires lifelong follow-up for surveillance.

According to the most recent classification of the World Health Organization, drawn up in 2022 and updated in 2024, ameloblastoma is classified into five clinical types: conventional (solid/multicystic), unicystic, adenoid, metastasizing, and peripheral/extraosseous [4,5].

Peripheral ameloblastoma (PA), also known as extraosseous ameloblastoma, soft tissue ameloblastoma, ameloblastoma of mucosal origin, or ameloblastoma of the gingiva, is a rare, unusual, benign, and extraosseous odontogenic tumor, which affects soft tissues and accounts for 1–10% of all ameloblastomas [2,6,7,8]. This term was first coined by Kuru in 1911, but the first complete description of this condition was provided by Stanley and Krogh in 1959 [2,6,8] and, until 2014, less than 200 cases of PA have been reported in the literature [6] (Table 1).

The 5th WHO histological classification of tumors of the oral cavity (2024) [5,9] included the extraosseous/peripheral ameloblastoma as one of the variants of ameloblastoma, suggesting that PA derives from the same histological structures related to the classic type. The tumor cell arises from the cell rest of Serres, the remnants of reduced enamel epithelium, and the basal cells of the surface epithelium [2,6].

However, differently from intraosseous ameloblastoma that presents at a younger age, PA arises between the third and the sixth decade of life (with an average age at presentation of 50 years old). PA is a rare tumor with a prevalence of 1–5% and mild male predilection (male/female ratio of 1.9:1); it occurs mainly in the gingival soft tissue [1,6,10,11,12].

Clinically, PA is a solid, painless, exophytic, extraosseous, sessile, and gradually growing lesion, arising from the soft tissue of tooth-bearing areas. It predominantly occurs in the gingiva (for this reason it is also named as “gingival ameloblastoma”) and it can be misinterpreted as other lesions with similar appearance, including pyogenic granuloma, peripheral ossifying fibroma, peripheral giant cell granuloma, and squamous papilloma. The lingual mandibular gingiva of the premolar area is the most involved site. This tumor does not generally invade the underlying bone; therefore, PA has been considered as a hamartomatous lesion, less aggressive than the conventional ameloblastoma [1,2,6,8,10,11,13].

The aim of this report is to describe the case of a middle-aged man diagnosed with peripheral ameloblastoma and to provide an updated overview of the current literature on this condition.

**Table 1:** Analysis of case reports and case series.

Author	Year	Patient, Age	Treatment	Follow-Up (Months)	Recurrence
Braunstein et al. [14]	1949	1 patient	Excision of soft-tissue lesion (blunt dissection)	4	No
Klinar et al. [15]	1969	1 patient	Surgical excision by extraoral approach, wide margins	2–3–5	No
Gardner et al. [16]	1977	21 patients	Excision (13 cases), electrocautery, extraction of teeth, removal of small amounts of bone, wide resection of the mandible with retention of the inferior border (1 case)	11–60	3
Patrikiou et al. [17]	1983	1 patient	Excision under general anesthesia with curettage of underlying bone	8	No
Atkinson et al. [18]	1984	10 patients	Megavoltage radiotherapy (4500 rads in 4 weeks) and 3 cases received adjuvant surgery	-	1
Schaberg et al. [19]	1985	1 patient	Excision with small margin of normal tissue, subsequent re-excision with larger margin of normal tissue	42	No
Buchner et al. [20]	1987	13 patients	Excision (initially incomplete in 4 cases but repeat excision performed)	-	1, peripheral ameloblastic carcinoma from recurrent lesion
Ficarra et al. [21]	1987	1 patient	Excision	60	No
Woo et al. [22]	1987	1 patient	Excision by intraoral approach	9	No
El-Mofty et al. [23]	1991	11 patients	Excision of the lesion down to the periosteum with small amount of normal tissue	-	1
Nauta et al. [24]	1992	Male, 32	Excision with an en bloc resection of the adjacent bone of the alveolar process	12	No
Hernandez et al. [25]	1992	1 patient	Excision down to level of bone (2 lesions)	24	1
Baden et al. [26]	1993	1 patient	Excision	60	2, ameloblastic carcinomas
Gurol et al. [27]	1995	8 patients	Complete excision through the periosteum without removing bone or teeth	3 cases with no follow-up reported, 2 cases 6 months, 1 case 24 m, 1 case 108 m, 1 case 120 m	No
Zhu et al. [28]	1995	1 patient	Excision including overlying gingiva and thin lingual alveolar bone	36	No
Califano et al. [29]	1996	1 patient	Surgical resection of the left maxilla with excision of bone surrounding the tumor	12	No
Wettan et al. [30]	2001	1 patient	Excision	36	2, with dysplastic change
Philipsen et al. [31]	2001	160 patients, 65% males and 35% female (male/female ratio 1.9:1), average age 52.1 years	Conservative supraperiosteal surgical excision with adequate disease-free margins	-	Recurrence rate 16–19%
Marucci et al. [32]	2004	1 patient	Radical surgical excision	-	-
Lopez-Jornet et al. [33]	2005	1 patient	Excisional biopsy with curettage of the affected mandibular bone	24	No
Martelli-Jùnior et al. [34]	2005	1 patient	Excision with narrow margin including underlying periosteum	12	No
Yanamoto et al. [35]	2005	1 patient	En bloc excision together with the maxillary canine and underlying alveolar bone, under local anesthesia; layer of exposed bony surface shaved with a round burr	180	No
Curtis et al. [36]	2006	1 patient	Resection of the lesion, buccal pad of fat and a mucosal flap for reconstruction	36	No
LeCorn et al. [37]	2006	1 patient	Excision	4	No
Gomes et al. [38]	2007	1 patient	Excisional biopsy	9	No
Yamanishi et al. [39]	2007	1 patient	Complete surgical excision by intraoral approach (blunt dissection)	7	No
Vanoven et al. [1]	2008	Man, 73	Resection of the tumor en bloc with partial maxillectomy with obturator reconstruction	-	No
Ide et al. [40]	2009	1 patient	Excision	12	No
Beena et al. [41]	2012	1 patient	Excisional biopsy of soft-tissue lesion	-	-
Bertossi et al. [42]	2014	1 patient	Resection of lesion with surrounding bone, extraction of the second molar, flap for closure	24	No
Bhat et al. [43]	2014	1 patient	Excised with a 5 mm margin using diathermy under general anesthesia	12	No
Lascane et al. [44]	2014	1 patient	Excisional biopsy under local anesthesia	12	No
Goda et al. [7]	2015	1 patient, female, 69	Complete surgical excision by intraoral approach (blunt dissection)	30	No
Borrello et al. [10]	2016	Female, 78	Excisional biopsy	12	No
Kandagal et al. [45]	2016	1 patient	Complete surgical excision of soft tissue lesion	24	No
Zhang et al. [8]	2018	25 patients, 22 males and 3 females (M:F = 7.3:1), average age 48.3 years (range 11–81 years, 1 case in pediatric patient)	Excision with or without partial bone resection	Periodic, every 6 months in the first 2 years and at least every 12 months, 3–180 (mean 61)	1, maybe due to incomplete removal of primary lesion
Janardhanan et al. [13]	2018	Male, 33	Excision	24	No
Nurik et al. [46]	2018	1 patient	Complete surgical excision	-	-
Upadhyaya et al. [47]	2018	18 patients, 6 females and 12 males	12 excisional biopsies, 5 incisional biopsies, 1 unknown	-	-
Cadavid et al. [48]	2019	2 patients	Treated conservatively with enucleation plus curettage or cryotherapy	120	No
On et al. [49]	2019	1 patient	Excisional biopsy of the lesion after the 5 cm incision and dissection of lateral wall of oropharynx by intraoral approach under general anesthesia	-	-
Ülker et al. [2]	2020	Female, 34	Excision	3	No
Vezhavendhan et al. [6]	2020	Male, 72	Excision	-	1, 6 years after the first excision

## 2. Materials and Methods

### Case Presentation

In November 2020, a 48-year-old Asian man was referred to the Oral Medicine Department (S.C. Odontostomatology II) of the St Paolo and Carlo Hospital in Milan because of a gingival growth at a right lower premolar area. The patient reported a previous diagnosis of PA of the lesion, made throughout incisional biopsy in another clinical unit.

On extraoral examination, no swelling, asymmetry, or lymphadenopathy were evident.

**Figure 1:**
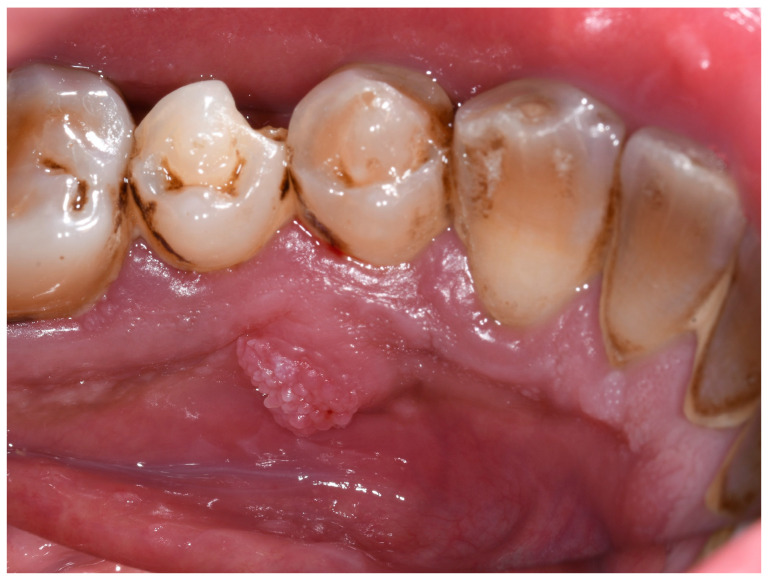
Intraoral photography: dome-shaped exophytic lesion with cobblestone surface and normal color, of 1 cm× 1 cm, located on the lingual aspect of the right mandibular premolar area.

Intraoral examination revealed a dome-shaped exophytic lesion, with cobblestone surface, and pink color, with a major axis of about 1 cm located on the lingual alveolar mucosa, in correspondence of tooth 4.4 (Figure 1). Tooth 4.4 was normoresponsive to vitality test, painless on percussion, and free from pathological periodontal probing, as were the adjacent teeth.

Periapical radiograph was taken, showing the absence of osseous alterations.

**Figure 2:**
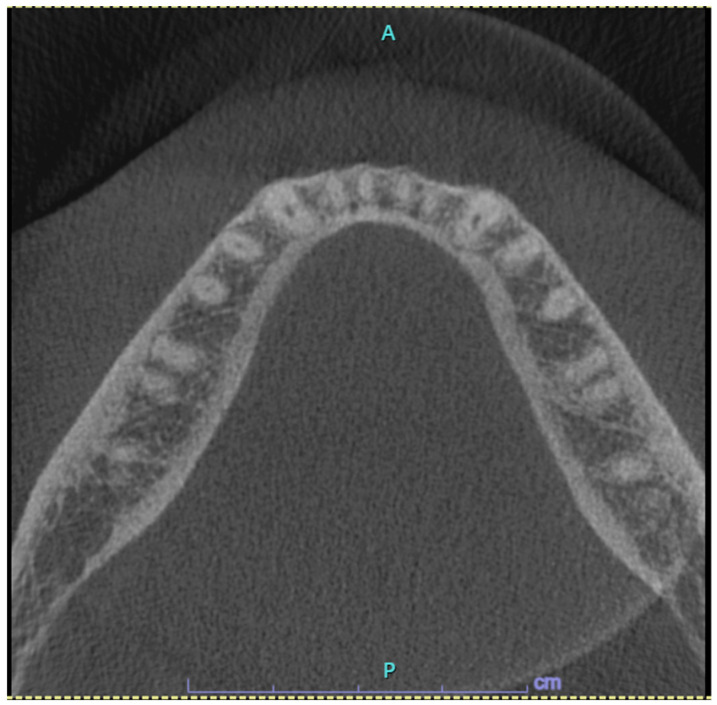
Axial Cone beam computed tomography (CBCT) sections showed the absence of altered density of trabecular bone, radiolucency, bone expansion, or changes in the mandibular lingual cortical bone in the right premolar area.

After patient’s informed consent, the diagnostic excisional biopsy of the lesion with histopathological investigation was performed, which confirmed the presence of PA. Axial Cone beam computed tomography (CBCT) scan revealed the absence of radiographic pathological alterations in the lingual mandibular cortical bone and of periosteal involvement and the absence of swelling at the site of the lesion (Figure 2).

Therefore, surgical excision of the lesion was carried out under local anesthesia with mepivacaine 2% with vasoconstrictor (adrenaline 1:100.000); non-absorbable 4/0 silk sutures were applied and hemostasis by compression was achieved. The specimen was fixed in formalin and sent to the Pathological Anatomy Department with a request for histopathological examination. No intraoperative and postoperative complications occurred. Postoperative instructions were provided and antiseptic and analgesic therapies were prescribed.

**Figure 3:**
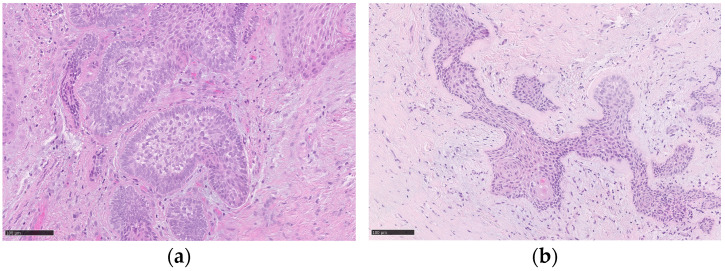
Histopathological findings show (**a**) the lesion involving the oral stroma, consisting of epithelial islands characterized by peripheral basal cells with nuclear palisade and reverse polarization of the nuclei; the cells present in the center of the islands are loosely arranged and have a lighter cytoplasm than that of the cells of the basal layer. (**b**) In other deeper islands, the aspects described previously are less evident and below, in the center of an island, a focus of squamous differentiation is present. The black scale bars represent, in both the images, 100 µm.

The 3-week follow-up visit showed the complete mucosal healing at the surgical site and the absence of recurrence of the lesion. Histopathological findings revealed the presence of a lesion consisting of epithelial islands characterized by peripheral basal cells with nuclear palisade and reverse polarization of the nuclei and central cells with a lighter cytoplasm that were loosely arranged. Furthermore, v-raf murine sarcoma viral oncogene homolog B (BRAF) immunohistochemistry was performed and it was negative. The histopathological report confirmed the presence of “mucosal fragment, coherent with oral/periodontal mucosa, with underlying multinodular odontogenic epithelial proliferation and resection margins in healthy tissue, with the morphologic features of peripheral ameloblastoma, with plexiform aspects and squamous metaplasia” (Figure 3).

**Figure 4:**
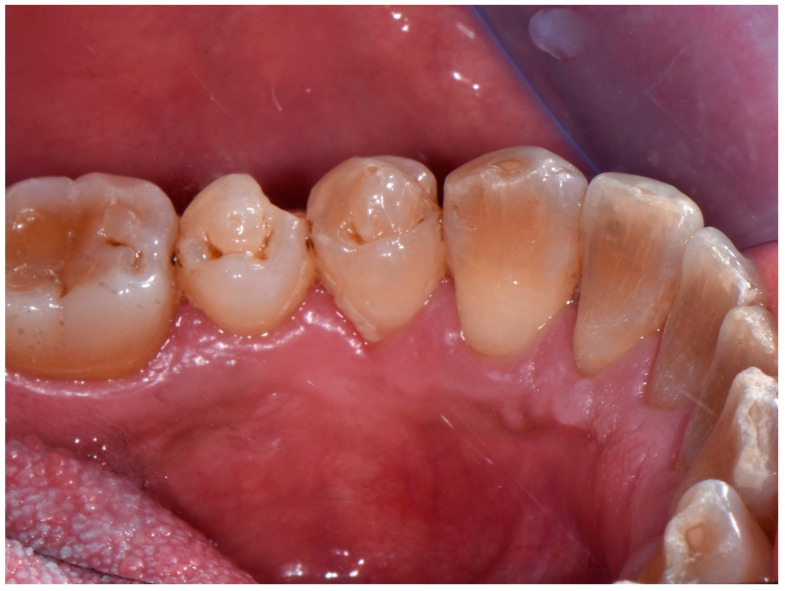
Clinical image showing the complete mucosal healing, without local recurrence.

At 6 months recall, the patient was still asymptomatic with oral mucosae free from pathological lesions; good healing of the surgical site and the absence of recurrence were observed (Figure 4).

**Figure 5:**
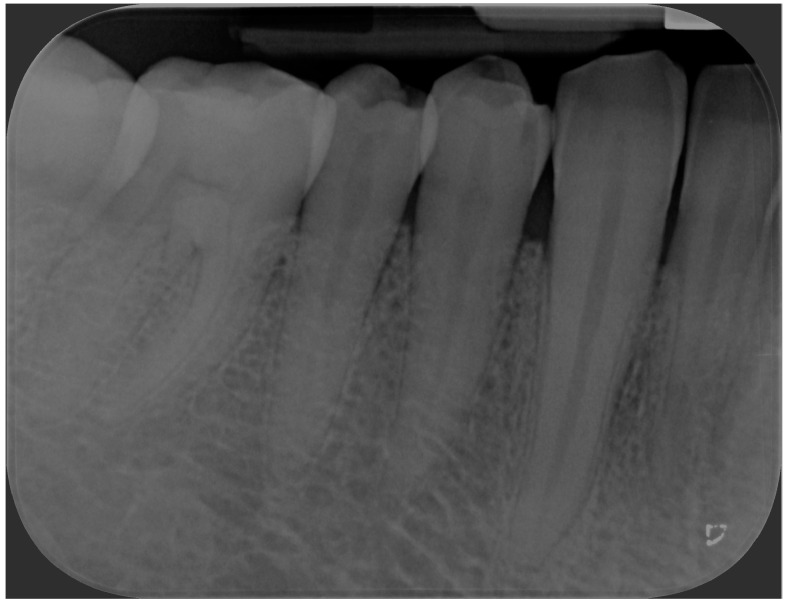
Periapical radiograph of the mandibular premolar right region after 6-months follow-up, showing no pathological alteration of the bone.

During the visit, a periapical radiograph of teeth 4.3, 4.4 and 4.5 was performed, from which no radiographic bone alterations of pathologic significance were evident (Figure 5).

**Figure 6:**
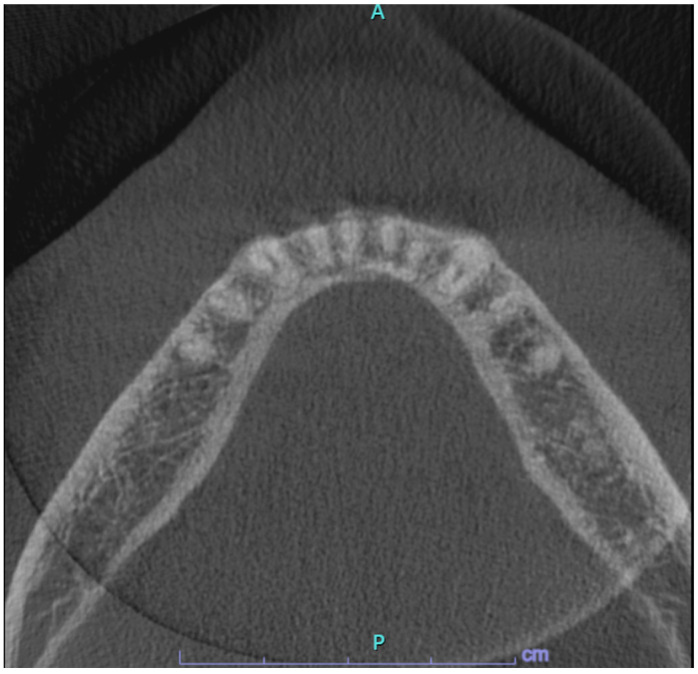
Axial CBCT sections at 2-years follow-up showing the absence of pathological changes.

Further follow-up examinations were scheduled at 1 and 2 years after the excisional biopsy for a clinical and radiological revaluation with CBCT scans in order to evaluate the integrity of the lingual cortical bone (Figure 6). The patient was asymptomatic and, at intraoral examination, no signs of recurrence could be observed: the mucosa showed a physiological appearance, pinkish and not ulcerated. The last follow-up after 3 years still showed clinical and radiographic stability of the picture. The patient is currently under active annual follow-up to early detect any recurrences.

## 3. Discussion

Ameloblastoma is the most frequent epithelial odontogenic tumor and accounts for about 11% of all odontogenic tumors [11]. According to the most recent classification of the World Health Organization, five subtypes exist: conventional (solid/multicystic), unicystic, metastasizing, adenoid, and peripheral ameblastomas (PA) [4]. Conventional ameloblastoma is the most common type of ameloblastoma, representing 57–63.8% of cases [50]; it mainly occurs in the mandible [51], without a clear sex predilection or ethnicity preference, in particular between the second and the fourth decade of life. Clinically, conventional ameloblastoma appears as a slow and asymptomatic cortical expansion; in cases of large dimension, tooth mobility, facial asymmetry, masticatory dysfunction, and pain can occur. Radiographic images include a multilocular radiolucent lesion, with well-defined and scalloped margins, sometimes also described as a “soap-bubble” appearance [52]. Differently from other types of ameloblastoma, the conventional type shows a more aggressive behavior and a higher recurrence rate; radical surgery is the most effective therapy [53]. Unicystic ameloblastoma is a rare variant of ameloblastoma, characterized by slow growth and relative local aggressiveness [54]. Radiographically, the typical finding is an expansive unilocular radiolucency with well-defined edges, sometimes associated with an impacted tooth [54]. Generally, the unicystic variant is considered less aggressive than the solid one; thus, the treatment of choice is enucleation or curettage, unless in the case of the unicystic ameloblastoma mural subtype, for which marginal resection is recommended [54]. The metastasizing type of ameloblastoma is an aggressive variant with the ability to metastasize mainly in the lungs and cervical lymph nodes [55]. Radiologically, there is a radiolucent/hypodense multiloculated lesion with irregular edges [55]. The first-choice treatment is a conservative surgery, associated with, when necessary, adjuvant therapies (such as radiation, chemotherapy, combination therapy, and neck dissection) [55]. Early and multiple recurrences, occurring post-treatment, are relatively common and they may signal a poor prognosis; for this reason, a long-term follow-up is necessary [55]. Adenoid ameloblastoma was recognized as a separate entity from the conventional ameloblastoma by the WHO in 2022, and it shows a slight predilection for females. The most common radiographic features are represented by ill-defined radiolucent and usually unilocular lesions, which can be associated with cortical perforation [56]. Histopathological findings include for this variant the presence of ductal structures, a cribriform architecture, epithelial whorls, and enamel knot-like structures; less often are found ghost cells and dentinoid [57]. This adenoid subtype has a locally aggressive behavior, with a high recurrence rate. Surgical resection with adequate disease-free margins and long-term follow-up are, also in this case, necessary [56].

PA, which is the subtype identified in our clinical case, is the rare extraosseous variant of ameloblastoma, characterized by a benign behavior and a minimal bony involvement; it represents 1–10% of all ameloblastomas [2,6,7,8]. Etiopathogenesis has been correlated with the cell rest of Serres, the remnants of reduced enamel epithelium, the basal cells of the surface epithelium, or with the pluripotent cells of minor salivary glands [6].

From a clinical point of view, PA appears as an exophytic slowly growing mass, either sessile or pedunculated, with a firm consistency [10,24,31]. The surface can be smooth, granular, warty, or papillary, and the color can range from normal mucosa to dark red [1]. The most affected intraoral site is the lingual gingiva of the canine/premolar region, followed by the anterior mandible and the maxillary tuber [6,24,31]. The maxilla is less involved than the mandible with an estimated ratio of 1:2.5 [6,24,31]. Extragingival localization is extremely rare, but some cases in the buccal mucosa, in the oral floor, and in the pterygomandibular space have been documented [6,24,31]. Even though the mass itself is usually painless, teeth migration can occur in case involving the interdental papilla [1].

The bone involvement of PA is absent or negligible, and the tumor usually remains superficial to the cortical bone [1,10], since the dense fibrous tissue of the gingiva and the periosteum may act as a barrier for the infiltration [8]. Nevertheless, a few cases in the literature have shown some bone involvement, named as “cupping” or “saucerization”, which appears as a small depression on the bone surface due to the local compression of the tumor [1,8]. For this reason, the use of 3D imaging, such as magnetic resonance (MRI) or computed tomography (CT), is useful to identify the lesion profile more precisely [11]. Our patient showed a pinkish-colored exophytic lesion, with papillary surface, localized on the lingual gingiva of the premolar region, without bone involvement or saucerization, as investigated by radiographic images.

Besides imaging, the final diagnosis of PA is made after the histopathologic examination [11,31,58], since the clinical presentation can be misdiagnosed with other conditions, such as pyogenic granuloma, fibrous epulis, peripheral ossifying fibroma, giant cell granuloma, inflammatory fibrous hyperplasia related to prosthesis, squamous papilloma, or intraoral basal cell carcinoma [1,2,59].

### 3.1. Differential Diagnosis of PA

Pyogenic granuloma is a benign reactive lesion, usually associated with chronic irritants such as plaque, calculus, or defective restoration margins. Clinically, it appears as a smooth or exophytic mass with a sessile or pedunculated base, characterized by different shades of color, from bright pink to red. The surface can sometimes be ulcerated, in particular in areas subjected to trauma. The most affected site is the gingiva, but it can also occur on the lips, buccal mucosa, and tongue. Pyogenic granuloma is usually asymptomatic, and the treatment of choice is surgical excision.

Fibrous epulis is another reactive lesion, which develops on the adherent gingiva as a response to food impaction, calculus, overhanging dental restorations, and other irritant factors. From a clinical point of view, it can be described as an exophytic, sessile, or pedunculated mass, with a more or less firm consistency and pink in color. The most frequent localization is at the interdental papilla, maybe due to the susceptibility of this area to the aggregation of food particles and plaque. The treatment consists in excising the lesion while removing local factors that can trigger its development.

Similarly, the peripheral ossifying fibroma (POF) is included in the differential diagnosis. It usually arises from interdental papilla and it shows a predilection for the anterior maxilla, especially incisor and canine areas. The histopathological findings show the presence of fibrous stroma in which mineralized tissues (such as bone and/or cementum-like) are present. Radiographs can show radiopaque opacification, occasionally associated with bone destruction.

The peripheral giant cell granuloma (PGCG) is a further reactive lesion in differential diagnosis; it is related to trauma or local irritation and it occurs mostly on the attached gingiva, on the alveolar mucosa, or on the crest of edentulous alveolar ridge. It appears as an exophytic mass, sessile or pedunculated, with a red and/or blue color, covered by a frequently ulcerated mucosa. Differently from the pyogenic granuloma, PGCG tends to affect in particular the molar area. The treatment includes complete surgical excision and the removal of causative factors.

The epulis fissuratum is an adaptive growth caused by chronic trauma and irritation from ill-fitting prosthesis. Considering its clinical features, it presents as a raised and sessile lesion in the form of folds, with a firm consistency. The overlying mucosa can be normal, erythematous, or ulcerated if it gets traumatized. The relining or remaking of the prosthesis and surgical excision are usually the choice treatment.

Squamous papilloma is one of the most common Human Papillomavirus (HPV)-related lesions, and it is mainly associated to genotype 6 and 11. Clinically, it appears as an exophytic growth with a characteristic warty surface, usually pedunculated, with color ranging from white to pink/red. The most affected intraoral sites are the palate, the tongue, and the labial mucosa. The average size of the squamous papilloma is normally less than 1 cm. Again, the therapy is the surgical removal of the lesion, also in order to decrease viral transmissibility.

Intraoral basal cell carcinoma is an extremely rare entity, which shares some common features with the PA. In fact, they both show a proliferation of basal cells, usually organized in nests, and intermixed with a fibrous stroma. Nevertheless, they can be distinguished relying on some microscopic and immunohistochemical findings: for example, the PA is positive for cytokeratin 19 and negative for Bcr-Ep4, while the opposite is seen in the intraoral basal cell carcinoma [1].

Considering its similarity, in terms of clinical aspect, to all the previous described conditions, the final diagnosis of PA is made after the histopathologic examination [2,11].

### 3.2. Histopathological Features of PA

Microscopically, PA is characterized by odontogenic epithelium organized in islands and chords, which show a follicular pattern and are similar to the odontogenic islands of the central ameloblastoma. Moreover, since the islands are found to be contiguous to the basal layer of the overlying surface epithelium, this arrangement can resemble the histopathological aspect of the basal cell carcinoma [4,6]. Considering the immunohistochemical features, PA shows a high positivity for cytokeratin 5, 14, and 19, as well as for calretinin and amelogenin; these findings may help the clinicians to rule out most of the odontogenic mesenchymal tumors, which are negative for these markers [1,6].

Immunohistochemistry may also highlight genetic mutation involved in the mitogen-activated protein kinase (MAPK) pathway, including BRAF, neuroblastoma RAS viral oncogene homolog (NRAS), and fibroblast growth factor receptor 2 (FGFR2) mutations. BRAF, in particular, is a member of the RAF kinase family and it plays an important role in the RAS-RAF-MAPK pathway, which regulates cell proliferation and differentiation. Overall, it is estimated that 80–90% of all subtypes of ameloblastoma are associated with the BRAF V600E mutation [60]. This latter occurs when valine (V) is substituted for glutamate (E) at codon 600. According to the current literature, the majority of ameloblastomas with this mutation are located in the mandible, in patients younger than 54 years old [61]. However, there is no statistically significant association according to histological variants or recurrence rate [61]. In our case, BRAF immunohistochemistry was was negative. Considering NRAS, a recent paper by Oh and Hong identified the NRAS G12D mutation in a case of peripheral ameloblastoma arising in the mandibular alveolar mucosa of a 65-year-old man [62].

### 3.3. Management of PA

The management of PA includes conservative local surgical excision with adequate disease-free margins, as preferential therapy [8,58]. However, due to the rarity of this condition, there is no consensus about the extent of the surgical margins [11]. After excision, the recurrence rate ranges from 16% to 19%, but some authors suggested that relapses might be attributed to incomplete excision of the primary lesion [6,11]. Although PA is considered a benign tumor, late recurrences (up to 10 years) or, more rarely, progressions to malignancy with recurrence as ameloblastic carcinoma have been documented [2,6,7,11]. In addition, a case of PA with malignant progression that also metastasized has been reported [1,7,46]. Therefore, a long-term periodic follow-up is highly suggested in order to detect any relapse or rare cases of malignant transformation [1,8]. 

Recently, with the elucidation of molecular markers of ameloblastoma, there have been attempts for the treatment of ameloblastoma with molecular targeted therapy, in particular used in patients with recurrent, metastatic, and malignant ameloblastic tumors [63,64]. Food and Drug Administration (FDA)-approved molecular targeted therapy for ameloblastomas include drugs able to inhibit the functions of mutated BRAF and mitogen-activated protein kinase kinase (MEK) [63,64].

## 4. Conclusions

PA is a rare variant of the classic and more common intraosseous counterpart. Considering its clinical aspect, which lacks pathognomonic features, the diagnosis is usually made after histopathological analysis, associated with 3D computed tomography (CT) imaging. The treatment of choice is surgical excision. Even though it is a benign lesion, recurrences or rare malignant transformations have been reported; therefore, the recommended patient’s management includes, besides the surgical excision of the lesion, a long-term follow-up.

## Data Availability

Data sharing is not applicable, since the article is a case report including a review of literature.
